# Potential Health Risks Associated to ICSI: Insights from Animal Models and Strategies for a Safe Procedure

**DOI:** 10.3389/fpubh.2014.00241

**Published:** 2014-11-17

**Authors:** María Jesús Sánchez-Calabuig, Angela Patricia López-Cardona, Raúl Fernández-González, Priscila Ramos-Ibeas, Noelia Fonseca Balvís, Ricardo Laguna-Barraza, Eva Pericuesta, Alfonso Gutiérrez-Adán, Pablo Bermejo-Álvarez

**Affiliations:** ^1^Departamento de Reproducción Animal, Instituto Nacional de Investigación y Tecnología Agraria y Alimentaria (INIA), Madrid, Spain; ^2^Departamento de Medicina y Cirugía Animal, Facultad de Veterinaria, Universidad Complutense de Madrid, Madrid, Spain

**Keywords:** ICSI, ART, IVF, DOHaD, animal models, sperm selection, transgenerational, imprinting

## Abstract

Artificial reproductive techniques are currently responsible for 1.7–4% of the births in developed countries and intracytoplasmatic sperm injection (ICSI) is the most commonly used, accounting for 70–80% of the cycles performed. Despite being an invaluable tool for infertile couples, the technique bypasses several biological barriers that naturally select the gametes to achieve an optimal embryonic and fetal development. In this perspective, ICSI has been associated with an increased risk for diverse health problems, ranging from premature births and diverse metabolic disorders in the offspring to more severe complications such as abortions, congenital malformations, and imprinting disorders. In this review, we discuss the possible implications of the technique *per se* on these adverse outcomes and highlight the importance of several experiments using mammalian models to truthfully test these implications and to uncover the molecular base that origins these health problems. We also dissect the specific hazards associated to ICSI and describe some strategies that have been developed to mimic the gamete selection occurring in natural conception in order to improve the safety of the procedure.

The great advances in artificial reproductive techniques (ART) over the last decades have fulfilled the dreams of millions of infertile couples. An estimated 3.75 million births have resulted from assisted conceptions (ESHRE 2010, ART fact sheet) and it has been estimated that 1.7–4% of all children born today in developed countries are conceived through the use of these techniques (1). Intracytoplasmatic sperm injection (ICSI) is currently the most commonly used ART, accounting to 70–80% of the cycles performed (2), and there is a trend toward an increase of its use worldwide (3), which highlights the importance of the study of the potential health risks associated with this technique.

The use of ICSI may pose a risk for the health of the mother and child. ARTs are considered a risk factor for different pregnancy complications such as high blood pressure, preeclampsia, growth retardation, bleeding or even premature births, and intrauterine death [reviewed in Ref. (4)], particularly, the risk of premature birth rises between two and three times depending on the study (5, 6). The health of the assisted-conception children may be also compromised. A recent consensus opinion review from a group of diverse experts working at ART clinics (the Evian Annual Reproduction Workshop Group) concluded that IVF/ICSI children have lower birthweights and higher peripheral fat, blood pressure, and fasting glucose concentrations than controls (7). Furthermore, multiple reports have associated more severe health problems such as congenital malformation or the appearance of imprinting disorders with the use of IVF/ICSI. The extensive literature available about the increased risk for congenital malformation in IVF/ICSI compared with naturally conceived children has been summarized in several meta-analyses. An analysis of 19 publications selected by a quality score based on sample size and appropriateness of control group observed that major malformation rates ranged from 0 to 9.5% in IVF, 1.1 to 9.7% for ICSI, and 0 to 6.9% in naturally conceived children, leading to a statistically significant overall odd ratio of 1.29 (8). A more recent review of 56 studies selected based on appropriateness of control group yielded an estimation for congenital malformations following IVF/ICSI of 1.37 compared with naturally conceived children (9). Large scale epidemiologic analyses have also observed an increased risk for congenital malformations following IVF/ICSI. A particularly sizeable study conducted in Israel, where the national insurance policy covers all IVF procedures for the first two children, reported an adjusted odd ratio for congenital malformations of 1.45 for the comparison between the IVF/ICSI population (9,042 live births) and naturally conceived infants (213,737 live births) (10). The odd ratio of this study was adjusted for other significant interacting factors such as maternal age or gender of the child, limiting the chance of spurious relation.

Genomic imprinting is an epigenetic mechanism based on DNA methylation at imprinting control regions (ICR) that determines the monoparental expression of a subset of genes. These methylation marks are established during gametogenesis in a sex-specific manner and remain unaltered after syngamia, evading the global demethylation taking place during preimplantation development (11). However, ART may alter this special protection resulting into abnormal imprinting patterns that lead to transcriptional dysregulation of imprinting genes. The altered transcriptional patterns of the imprinting genes leads to aberrant embryonic and placental development, ultimately manifested as imprinting syndromes in the offspring (12). Several of these syndromes, such as Beckwith–Wiedemann (BWS), Angelman (AS), Silver–Russel syndrome, and retinoblastoma have been associated with ART, but others negate this association [reviewed in Ref. (13)]. One comprehensive meta-analysis suggested that only three imprinting disorders BWS, AS, and maternal hypomethylation syndrome, all of which associated with hypomethylation at different maternal ICRs, have been consistently linked to the use of ART (14). Later epidemiologic observations regarding BWS and AS also agree with the notion that an ART-induced hypomethylation at ICR is responsible for the increased incidence of imprinting disorders in IVF/ICSI children. BWS and AS may have a genetic (i.e., mutations in the DNA sequence) or epigenetic (i.e., imprinting defect: alterations in the methylation patterns at ICRs) origin. It has been observed that 90–100% of the IVF/ICSI children with BWS had imprinting defects, in contrast with 40–50% of the naturally conceived children with BWS (15). Likewise, whereas only 5% of the spontaneously conceived children with AS had an epigenetic origin, 71% of the AS cases in IVF/ICSI children were attributed to imprinting defects (15). In a similar context, a lower DNA methylation has been reported in the placenta of children conceived *in vitro* compared to the control, although the reduction in CpG methylation affected equally imprinted and non-imprinted regions (16).

## Put the Blame on ICSI?

Some of the adverse outcomes observed following ART may be due to the increased risk for health problems in couples pursuing ART, rather than the ART *per se* (9, 17, 18). The contribution of this parental factor to the health problems associated to the ART is difficult to dissect from the risk derived from the technique itself in human studies, as the appropriate control group to establish a possible relation between ICSI and risk of pregnancy complications or birth defects should be babies naturally conceived by infertile couples. Also, the rarity of the some of the diseases associated to ART such as congenital malformations or imprinting disorders result in a very low statistical power (17). Unfertile couples often show an increased risk factor for pregnancy disorders. Women are on average older, which increases the proportion of low quality oocytes with chromosome abnormalities (19), however, even with donor oocytes, only 5% of fresh oocytes produce a baby (20). Furthermore, other potential risk factors such as cycle irregularities, uterine anomalies, or obesity – with mixed effects on oocyte quality or uterine receptivity (21) – are also more common in these patients (4). The higher occurrence of multiple pregnancies resulting from the transfer of more than one embryo constitutes another risk factor for pregnancy complications (4). On the paternal side, the spermatozoa from infertile male have been shown to display genetic and epigenetic alterations that can be linked to a reduced embryo development and the appearance of abnormal phenotypes in their offspring. In subfertile men, a higher incidence of DNA fragmentation (22, 23) and aberrant DNA methylation at ICRs (24) have been reported.

**Figure 1:**
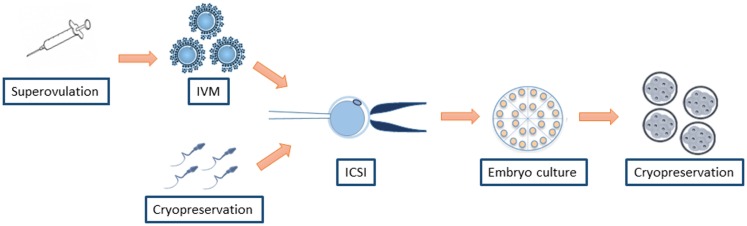
**ART associated to ICSI that may play a role in increasing the risk for health problems**.

On the other hand, it is difficult to discern between the damage associated to ICSI and other ARTs associated to the procedure. The assisted reproduction treatment for ICSI is not limited to the injection of a sperm head into an oocyte. It also involves the hormonal induction to achieve supernumerary oocyte production, *in vitro* maturation (IVM) of the oocytes retrieved, *in vitro* culture (IVC) of the zygotes produced by ICSI, and cryopreservation of gametes and embryos, all of which may play a role in hampering optimal embryo development (25, 26) (Figure 1). In this context, IVF and ICSI share most of the procedures but the sperm injection, which evades the spermatozoa selection at the zona pellucida (ZP). The possible differential risk for adverse outcomes between both procedures remains controversial. Experiments conducted in animal models have observed several developmental alterations exclusively attributed to ICSI, as detailed below. Besides, an epidemiologic study reported a higher major malformation rate in babies obtained by ICSI with cryopreserved sperm compared with IVF (8.4 vs. 4.6%) (27). However, large meta-analyses of a mix of selected publications using fresh or frozen spermatozoa under different conditions did not observe a differential risk for birth defects between ICSI and IVF (8, 9).

## Negative Effects of ICSI Observed in Mammalian Models

Mammalian models constitute a valuable tool to study the adverse outcomes associated with ART. Studies in animal models provide a proper control group of healthy and fertile animals, reduce the environmental variations, and provide a pre-set experimental frame that avoids selective reporting. Furthermore, the molecular mechanisms behind the phenotypic alterations caused by ART are shared between mammalian species. A very well-known example of this is the so called “large offspring syndrome” in ruminants, caused by suboptimal IVC, remarkably similar in phenotype and molecular base to the human BWS (28).

Due to practical and technical limitations in ruminants or pigs, the rodents have been the most frequently used models to study the long-term effects of ICSI in the health of the offspring. In rodent models, ICSI has been reported to alter DNA decondensation (29) and calcium oscillation (30) in mouse zygotes compared with IVF, and to impair the active demethylation of the male pronucleus in rat zygotes (31). ICSI performed with fresh sperm has been reported to increase the appearance of abnormal chromosome segregation (ACS) at the first mitotic division in mouse (32). Half of these ACS embryos developed into morphologically normal blastocysts able to implant, but unable to develop to term, resulting in spontaneous abortions at E7.5 (32). However, less severely impaired, yet abnormal embryos produced by ICSI may be able to survive through pregnancy, resulting in long-term effects on the adult life in the context of the Developmental Origins of Adult Health and Disease (DOHaD). In this perspective, several alterations have been described in the offspring, such as aberrant transcriptional aberrations spanning to the neonatal stage (33), alterations in glucose parameters in adult mice (34), and decreased testis weight, abnormal testicular tubule morphology, and increased testicular apoptosis (35). These long-term effects are usually manifested in a sex-specific manner, which can be explained by the widespread epigenetic sexual dimorphism observed in preimplantation embryos (36–38).

The situation is more severe when DNA-fragmented spermatozoa are used for ICSI (DFS-ICSI). The study of DFS-ICSI effects in animal models is particularly relevant because a significant proportion of infertile men have elevated levels of DNA damage in their ejaculated spermatozoa, which may be morphologically normal and thereby inadvertently used for ICSI (39). Sperm DNA fragmentation has been reported to affect embryo post-implantation development in ICSI procedures in humans, resulting in pregnancy loss (40). Studies in mice using DFS-ICSI, produced by freeze-thawing without cryoprotectants, have observed that DFS-ICSI induces epigenetic and genetic alterations in the embryo, resulting in detrimental effects in the offspring. On preimplantation mouse embryos, DFS-ICSI has been observed to delay male pronucleus demethylation, alter blastocyst gene expression, and modify the expression of imprinting genes (41). These early alterations may result in embryonic death or in aberrant phenotypes in the offspring, such as aberrant growth, premature aging, abnormal behavior, and a higher incidence of mesenchymal tumors (41). Furthermore, male offspring produced by DFS-ICSI has been reported to display a reduced fertility (42). The same study also observed that the offspring derived from DFS-ICSI displayed an increased chance for the appearance of abnormal phenotypes (kinky-tail) in the *Axin^Fu^* mouse model of metastable epiallele, suggesting a transgenerational inheritance of the epigenetic alterations generated by DFS-ICSI (42). However, the heritability of other epigenetic changes has not been observed in other studies (43), so the transgenerational epigenetic inheritance may depend on the specific epigenetic alteration.

## ICSI-Specific Hazards: Bypassing Natural Barriers

While dissecting the ICSI-specific hazards, two are exclusive from this technique: the injection of the sperm head into the oocyte and the bypass of the natural spermatozoa selection mechanisms, which is partly shared with IVF.

The micromanipulation itself may harm the oocyte and the resulting embryo. Oocyte activation seems to be abnormal, as Ca^2+^ oscillations differ between mouse IVF and ICSI (30). The injection of a sperm head that has not undergone acrosome reaction increases the risk of vacuole formation in the oocyte. Although this effect, observed in mice, only happened when three or more intact spermatozoa were injected (44), removal of sperm plasma membrane improved embryo development in mice (45) and selection for spermatozoa with reacted acrosome improved implantation rates in humans (46) and mouse (29).

**Figure 2:**
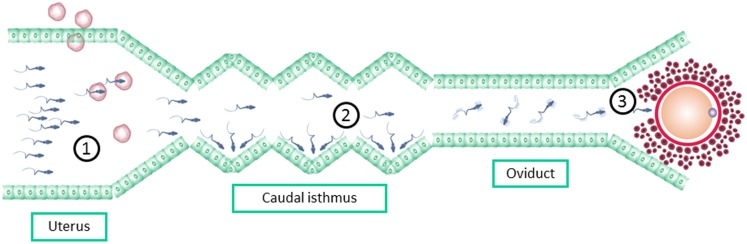
**Spermatozoa selection barriers bypassed by ICSI: (1) the female reproductive tract microenvironment, including immune cells (2) the sperm–oviduct interactions at the caudal isthmus, and (3) the sperm–zone pellucida interaction**.

Although a large number of spermatozoa are present in the ejaculate, only a minority reach the fertilization place. On the journey of the sperm from the site of deposition to the site of fertilization, spermatozoa should pass through different barriers that ensure that only those with normal morphology and vigorous motility will have chances to fertilize the oocyte forming a healthy embryo (47, 48). Spermatozoa selection under natural circumstances is based on three different steps: (1) the female reproductive tract microenvironment, (2) the sperm–oviduct interactions at the caudal isthmus, and (3) the sperm–zone pellucida interaction (48) (Figure 2). ICSI bypasses this natural selection process of the fertilizing sperm (49), as it does not allow the sperm–oviduct interaction and other spermatozoa selection processes including ZP binding-penetration.

Spermatozoa DNA fragmentation is one of the most studied spermatozoa alterations generally excluded by these bypassed natural barriers. Furthermore, membrane altered spermatozoa have been reported to release endonucleases to the media that could induce DNA fragmentation in spermatozoa with unaltered membranes (50). Female genital tract seems to act as a selective barrier against DNA fragmentation. A study in the mouse model showed that natural mating with males showing an increased percentage of spermatozoa DNA damage – produced by scrotal heat treatment or irradiation – resulted in different percentages of DNA fragmentation according to the region of the female reproductive tract where the sample was collected. Particularly, those spermatozoa reaching the oviduct had lower DNA fragmentation compared with those situated on lower, more distant to the fertilization place, portions (51). Apart from this selection at the reproductive tract, the study also pointed spermatozoa binding to the ZP as a second barrier against DNA-fragmented spermatozoa, as the percentage of DNA fragmentation was lower for the sperm bound to the ZP than to the unattached spermatozoa (51).

Since it is unlikely that uterine or oviductal cells are able to assess sperm DNA quality *per se*, the selection needs to be based on sperm phenotype and function related to DNA integrity (52). Among the possible candidates, motility is the main selective factor on the trip (53), but it is not the only one, as other barriers such as leukocytic/phagocytic responses from the immune cells present in the uterine mucosa (54) may play a role, and binding to the oviduct has been correlated with chromatin stability in the pig model (55). Some studies have found a correlation between DNA integrity and sperm motility. Thus, a negative correlation was established between the computer-aided sperm analysis (CASA) percentage of motile sperms and DNA fragmentation index (DFI) (56). Likewise, negative correlations were observed between sperm DNA fragmentation assayed by TUNEL and sperm motility under natural conditions (57) or after H_2_O_2_ or alpha irradiation-mediated DNA damage (58). In agreement, the spermatozoa selection method swim-up, which enriches for motile spermatozoa, reduces the percentage of apoptotic spermatozoa (59).

On a similar context, the binding of spermatozoa to ZP selects those with progressive motility, normal morphology, and chromatin structure (60). Spermatozoa with single stranded or denatured DNA were reported to bind less or do not bind at all to the ZP (61). ZP binding may even exclude those with numerical chromosomal aberrations (62), which seem not to display impaired motility (63).

## Strategies for a Safer ICSI

As the bypass of spermatozoa selection seems to be one of the most critical hazards when performing ICSI, several spermatozoa selection methods have been proposed. Density gradients commonly used in IVF are able to separate dead and alive spermatozoa and other techniques such as Swim-up allow the selection of motile spermatozoa, less prone to display DNA fragmentation (59). Similarly, novel methods for spermatozoa selection based on motility have been developed in microfluidics platforms, allowing sperm selection in oligozoospermic samples with high amounts of non-gamete cell contamination (64), and enriching the sample for sperm with intact chromatin and DNA integrity (65). High resolution morphology has also been used to improve implantation rates (66). In this line, motile sperm organelle morphology examination (MSOME) allows grading spermatozoa based on the detection of vacuoles in the sperm heads (67). These vacuoles negatively affect implantation, pregnancy, and live birth rates following ICSI (68). However, the beneficial effects of applying morphological criteria for sperm selection before ICSI on implantation and pregnancy rates remain a controversial issue, with contrasting results obtained by different groups [reviewed in Ref. (69)]. Other techniques such as the use of sperm selection chambers (70), a peptide ligand based stain capable of binding damaged DNA structures (71), and Raman microspectroscopy (72) have been proposed to select spermatozoa with low DNA damage, whereas birefringency was used to select acrosome reacted spermatozoa (46). In a similar line, hyaluronic acid sperm selection or ZP binding before ICSI have been reported to be able to select for mature spermatozoa, reduce DNA fragmentation rate, and improve embryo quality and development (73–75). These binding-based techniques have been suggested to select against immature sperm that has not reached its final nuclear and cytoplasmic maturation (76).

Oocyte quality is another factor to take into account, as on one hand the oocyte itself may be the source for genetic or epigenetic alterations (77, 78) and on the other it may repair the genetic or epigenetic alterations of the spermatozoa. Oocytes have been suggested to be able to repair sperm DNA when the damage is <8% (79), but the DNA repair ability depends on oocyte quality and age (78). Thereby, improvements in IVM or ovarian stimulation may reduce the adverse effects of ICSI. In this sense, the use of low hormone doses may help toward a more stringent selection of oocytes (7).

## Concluding Remarks

The current widespread use of ICSI together with the perspective of growth in its use urge for an analysis of the possible health risk associated with this technique. Epidemiologic studies have established associations between the use of ICSI and diverse health problems, ranging from premature births and metabolic complications in the offspring to abortions, congenital malformations, and imprinting disorders. Animal models provide an invaluable means to experimentally test these associations and to understand the molecular root behind the adverse outcomes of ICSI in the offspring, as an initial step to improve the safety of the technique. ICSI bypasses a series of biological barriers, but novel strategies based on gamete selection may mimic these barriers, restoring the natural selection process required for a flawless embryonic and fetal development.

## Conflict of Interest Statement

The authors declare that the research was conducted in the absence of any commercial or financial relationships that could be construed as a potential conflict of interest.

## References

[1] Williams C Sutcliffe A Infant outcomes of assisted reproduction. Early Hum Dev (2009) 85(11):673–7 10.1016/j.earlhumdev.2009.08.055 19740614

[2] Palermo GD Neri QV Takeuchi T Rosenwaks Z. ICSI: where we have been and where we are going. Semin Reprod Med (2009) 27(2):191–201. 10.1055/s-0029-1202309 19247922

[3] Nyboe Andersen A Carlsen E Loft A. Trends in the use of intracytoplasmatic sperm injection marked variability between countries. Hum Reprod Update (2008) 14(6):593–604. 10.1093/humupd/dmn032 18708651

[4] Zollner U Dietl J Perinatal risks after IVF and ICSI. J Perinat Med (2013) 41(1):17–22 10.1515/jpm-2012-0097 23095186

[5] Helmerhorst FM Perquin DA Donker D Keirse MJ. Perinatal outcome of singletons and twins after assisted conception: a systematic review of controlled studies. BMJ (2004) 328(7434):261. 10.1136/bmj.37957.560278.EE 14742347 PMC324454

[6] Schieve LA Meikle SF Ferre C Peterson HB Jeng G Wilcox LS. Low and very low birth weight in infants conceived with use of assisted reproductive technology. N Engl J Med (2002) 346(10):731–7. 10.1056/NEJMoa010806 11882728

[7] Fauser BC Devroey P Diedrich K Balaban B Bonduelle M Delemarre-van de Waal HA Health outcomes of children born after IVF/ICSI: a review of current expert opinion and literature. Reprod Biomed Online (2014) 28(2):162–82. 10.1016/j.rbmo.2013.10.013 24365026

[8] Rimm AA Katayama AC Diaz M Katayama KP. A meta-analysis of controlled studies comparing major malformation rates in IVF and ICSI infants with naturally conceived children. J Assist Reprod Genet (2004) 21(12):437–43. 10.1007/s10815-004-8760-8 15704519 PMC3455612

[9] Wen J Jiang J Ding C Dai J Liu Y Xia Y Birth defects in children conceived by in vitro fertilization and intracytoplasmic sperm injection: a meta-analysis. Fertil Steril (2012) 97(6):1331–7. 10.1016/j.fertnstert.2012.02.053 22480819

[10] Farhi A Reichman B Boyko V Mashiach S Hourvitz A Margalioth EJ Congenital malformations in infants conceived following assisted reproductive technology in comparison with spontaneously conceived infants. J Matern Fetal Neonatal Med (2013) 26(12):1171–9. 10.3109/14767058.2013.776535 23451839

[11] Smallwood SA Tomizawa S Krueger F Ruf N Carli N Segonds-Pichon A Dynamic CpG island methylation landscape in oocytes and preimplantation embryos. Nat Genet (2011) 43(8):811–4. 10.1038/ng.864 21706000 PMC3146050

[12] Young LE Fernandes K McEvoy TG Butterwith SC Gutierrez CG Carolan C Epigenetic change in IGF2R is associated with fetal overgrowth after sheep embryo culture. Nat Genet (2001) 27(2):153–4. 10.1038/84769 11175780

[13] Eroglu A Layman LC. Role of ART in imprinting disorders. Semin Reprod Med (2012) 30(2):92–104. 10.1055/s-0032-1307417 22549709 PMC3838883

[14] Amor DJ Halliday J. A review of known imprinting syndromes and their association with assisted reproduction technologies. Hum Reprod (2008) 23(12):2826–34. 10.1093/humrep/den310 18703582

[15] Manipalviratn S DeCherney A Segars J Imprinting disorders and assisted reproductive technology. Fertil Steril (2009) 91(2):305–15 10.1016/j.fertnstert.2009.01.002 19201275 PMC3081604

[16] Katari S Turan N Bibikova M Erinle O Chalian R Foster M DNA methylation and gene expression differences in children conceived in vitro or in vivo. Hum Mol Genet (2009) 18(20):3769–78. 10.1093/hmg/ddp319 19605411 PMC2748887

[17] Vermeiden JP Bernardus RE. Are imprinting disorders more prevalent after human in vitro fertilization or intracytoplasmic sperm injection? Fertil Steril (2013) 99(3):642–51. 10.1016/j.fertnstert.2013.01.125 23714438

[18] Hansen M Kurinczuk JJ Milne E de Klerk N Bower C Assisted reproductive technology and birth defects: a systematic review and meta-analysis. Hum Reprod Update (2013) 19(4):330–53 10.1093/humupd/dmt006 23449641

[19] Balasch J Gratacos E. Delayed childbearing: effects on fertility and the outcome of pregnancy. Curr Opin Obstet Gynecol (2012) 24(3):187–93. 10.1097/GCO.0b013e3283517908 22450043

[20] Patrizio P Sakkas D. From oocyte to baby: a clinical evaluation of the biological efficiency of in vitro fertilization. Fertil Steril (2009) 91(4):1061–6. 10.1016/j.fertnstert.2008.01.003 18325517

[21] Bermejo-Alvarez P Rosenfeld CS Roberts RM. Effect of maternal obesity on estrous cyclicity, embryo development and blastocyst gene expression in a mouse model. Hum Reprod (2012) 27(12):3513–22. 10.1093/humrep/des327 23001779 PMC3501243

[22] Irvine DS Twigg JP Gordon EL Fulton N Milne PA Aitken RJ DNA integrity in human spermatozoa: relationships with semen quality. J Androl (2000) 21(1):33–44 10.1093/humrep/dei231 10670517

[23] Sergerie M Laforest G Bujan L Bissonnette F Bleau G. Sperm DNA fragmentation: threshold value in male fertility. Hum Reprod (2005) 20(12):3446–51. 10.1093/humrep/dei231 16085665

[24] Sato A Hiura H Okae H Miyauchi N Abe Y Utsunomiya T Assessing loss of imprint methylation in sperm from subfertile men using novel methylation polymerase chain reaction Luminex analysis. Fertil Steril (2011) 95(1):129–34. 10.1016/j.fertnstert.2010.06.076 20655520

[25] Talaulikar VS Arulkumaran S. Maternal, perinatal and long-term outcomes after assisted reproductive techniques (ART): implications for clinical practice. Eur J Obstet Gynecol Reprod Biol (2013) 170(1):13–9. 10.1016/j.ejogrb.2013.04.014 23759305

[26] Fernandez-Gonzalez R Moreira P Bilbao A Jimenez A Perez-Crespo M Ramirez MA Long-term effect of in vitro culture of mouse embryos with serum on mRNA expression of imprinting genes, development, and behavior. Proc Natl Acad Sci U S A (2004) 101(16):5880–5. 10.1073/pnas.0308560101 15079084 PMC395892

[27] Belva F Henriet S Van den Abbeel E Camus M Devroey P Van der Elst J Neonatal outcome of 937 children born after transfer of cryopreserved embryos obtained by ICSI and IVF and comparison with outcome data of fresh ICSI and IVF cycles. Hum Reprod (2008) 23(10):2227–38. 10.1093/humrep/den254 18628260

[28] Robbins KM Chen Z Wells KD Rivera RM. Expression of KCNQ1OT1, CDKN1C, H19, and PLAGL1 and the methylation patterns at the KvDMR1 and H19/IGF2 imprinting control regions is conserved between human and bovine. J Biomed Sci (2012) 19:95. 10.1186/1423-0127-19-95 23153226 PMC3533950

[29] Ajduk A Yamauchi Y Ward MA. Sperm chromatin remodeling after intracytoplasmic sperm injection differs from that of in vitro fertilization. Biol Reprod (2006) 75(3):442–51. 10.1095/biolreprod.106.053223 16775225

[30] Kurokawa M Fissore RA. ICSI-generated mouse zygotes exhibit altered calcium oscillations, inositol 1,4,5-trisphosphate receptor-1 down-regulation, and embryo development. Mol Hum Reprod (2003) 9(9):523–33. 10.1093/molehr/gag072 12900511

[31] Yoshizawa Y Kato M Hirabayashi M Hochi S. Impaired active demethylation of the paternal genome in pronuclear-stage rat zygotes produced by in vitro fertilization or intracytoplasmic sperm injection. Mol Reprod Dev (2010) 77(1):69–75. 10.1002/mrd.21109 19743475

[32] Yamagata K Suetsugu R Wakayama T. Assessment of chromosomal integrity using a novel live-cell imaging technique in mouse embryos produced by intracytoplasmic sperm injection. Hum Reprod (2009) 24(10):2490–9. 10.1093/humrep/dep236 19574276

[33] Kohda T Ogonuki N Inoue K Furuse T Kaneda H Suzuki T Intracytoplasmic sperm injection induces transcriptome perturbation without any transgenerational effect. Biochem Biophys Res Commun (2011) 410(2):282–8. 10.1016/j.bbrc.2011.05.133 21658372

[34] Scott KA Yamazaki Y Yamamoto M Lin Y Melhorn SJ Krause EG Glucose parameters are altered in mouse offspring produced by assisted reproductive technologies and somatic cell nuclear transfer. Biol Reprod (2010) 83(2):220–7. 10.1095/biolreprod.109.082826 20445127 PMC2907285

[35] Yu Y Zhao C Lv Z Chen W Tong M Guo X Microinjection manipulation resulted in the increased apoptosis of spermatocytes in testes from intracytoplasmic sperm injection (ICSI) derived mice. PLoS One (2011) 6(7):e22172. 10.1371/journal.pone.0022172 21799787 PMC3140508

[36] Bermejo-Alvarez P Rizos D Rath D Lonergan P Gutierrez-Adan A. Epigenetic differences between male and female bovine blastocysts produced in vitro. Physiol Genomics (2008) 32(2):264–72. 10.1152/physiolgenomics.00234.2007 17986520

[37] Bermejo-Alvarez P Rizos D Rath D Lonergan P Gutierrez-Adan A. Sex determines the expression level of one third of the actively expressed genes in bovine blastocysts. Proc Natl Acad Sci U S A (2010) 107(8):3394–9. 10.1073/pnas.0913843107 20133684 PMC2840439

[38] Bermejo-Alvarez P Rizos D Lonergan P Gutierrez-Adan A. Transcriptional sexual dimorphism during preimplantation embryo development and its consequences for developmental competence and adult health and disease. Reproduction (2011) 141(5):563–70. 10.1530/REP-10-0482 21339284

[39] Zini A Meriano J Kader K Jarvi K Laskin CA Cadesky K. Potential adverse effect of sperm DNA damage on embryo quality after ICSI. Hum Reprod (2005) 20(12):3476–80. 10.1093/humrep/dei266 16123087

[40] Borini A Tarozzi N Bizzaro D Bonu MA Fava L Flamigni C Sperm DNA fragmentation: paternal effect on early post-implantation embryo development in ART. Hum Reprod (2006) 21(11):2876–81. 10.1093/humrep/del251 16793992

[41] Fernandez-Gonzalez R Moreira PN Perez-Crespo M Sanchez-Martin M Ramirez MA Pericuesta E Long-term effects of mouse intracytoplasmic sperm injection with DNA-fragmented sperm on health and behavior of adult offspring. Biol Reprod (2008) 78(4):761–72. 10.1095/biolreprod.107.065623 18199884

[42] Ramos-Ibeas P Calle A Fernandez-Gonzalez R Laguna-Barraza R Pericuesta E Calero A Intracytoplasmic sperm injection using DNA-fragmented sperm in mice negatively affects embryo-derived embryonic stem cells, reduces the fertility of male offspring and induces heritable changes in epialleles. PLoS One (2014) 9(4):e95625 10.1371/journal.pone.0095625 24743851 PMC3990723

[43] de Waal E Yamazaki Y Ingale P Bartolomei M Yanagimachi R McCarrey JR. Primary epimutations introduced during intracytoplasmic sperm injection (ICSI) are corrected by germline-specific epigenetic reprogramming. Proc Natl Acad Sci U S A (2012) 109(11):4163–8. 10.1073/pnas.1201990109 22371603 PMC3306712

[44] Morozumi K Yanagimachi R Incorporation of the acrosome into the oocyte during intracytoplasmic sperm injection could be potentially hazardous to embryo development. Proc Natl Acad Sci U S A (2005) 102(40):14209–14 10.1073/pnas.0507005102 16183738 PMC1242329

[45] Morozumi K Shikano T Miyazaki S Yanagimachi R. Simultaneous removal of sperm plasma membrane and acrosome before intracytoplasmic sperm injection improves oocyte activation/embryonic development. Proc Natl Acad Sci U S A (2006) 103(47):17661–6. 10.1073/pnas.0608183103 17090673 PMC1693803

[46] Gianaroli L Magli MC Ferraretti AP Crippa A Lappi M Capitani S Birefringence characteristics in sperm heads allow for the selection of reacted spermatozoa for intracytoplasmic sperm injection. Fertil Steril (2010) 93(3):807–13. 10.1016/j.fertnstert.2008.10.024 19064263

[47] Barratt CL Kirkman-Brown J. Man-made versus female-made environment – will the real capacitation please stand up? Hum Reprod Update (2006) 12(1):1–2. 10.1093/humupd/dmi051 16354709

[48] Suarez SS Pacey AA Sperm transport in the female reproductive tract. Hum Reprod Update (2006) 12(1):23–37 10.1093/humupd/dmi047 16272225

[49] Schultz RM Williams CJ The science of ART. Science (2002) 296(5576):2188–90 10.1126/science.1071741 12077406

[50] Perez-Crespo M Moreira P Pintado B Gutierrez-Adan A. Factors from damaged sperm affect its DNA integrity and its ability to promote embryo implantation in mice. J Androl (2008) 29(1):47–54. 10.2164/jandrol.107.003194 17673434

[51] Hourcade JD Perez-Crespo M Fernandez-Gonzalez R Pintado B Gutierrez-Adan A. Selection against spermatozoa with fragmented DNA after postovulatory mating depends on the type of damage. Reprod Biol Endocrinol (2010) 8:9. 10.1186/1477-7827-8-9 20113521 PMC2825232

[52] Holt WV Van Look KJ. Concepts in sperm heterogeneity, sperm selection and sperm competition as biological foundations for laboratory tests of semen quality. Reproduction (2004) 127(5):527–35. 10.1530/rep.1.00134 15129008

[53] Nakanishi T Isotani A Yamaguchi R Ikawa M Baba T Suarez SS Selective passage through the uterotubal junction of sperm from a mixed population produced by chimeras of calmegin-knockout and wild-type male mice. Biol Reprod (2004) 71(3):959–65. 10.1095/biolreprod.104.028647 15151931

[54] Schuberth HJ Taylor U Zerbe H Waberski D Hunter R Rath D. Immunological responses to semen in the female genital tract. Theriogenology (2008) 70(8):1174–81. 10.1016/j.theriogenology.2008.07.020 18757083

[55] Ardon F Helms D Sahin E Bollwein H Topfer-Petersen E Waberski D. Chromatin-unstable boar spermatozoa have little chance of reaching oocytes in vivo. Reproduction (2008) 135(4):461–70. 10.1530/REP-07-0333 18367507

[56] Giwercman A Richthoff J Hjollund H Bonde JP Jepson K Frohm B Correlation between sperm motility and sperm chromatin structure assay parameters. Fertil Steril (2003) 80(6):1404–12 10.1016/S0015-0282(03)02212-X 14667876

[57] Huang CC Lin DP Tsao HM Cheng TC Liu CH Lee MS. Sperm DNA fragmentation negatively correlates with velocity and fertilization rates but might not affect pregnancy rates. Fertil Steril (2005) 84(1):130–40. 10.1016/j.fertnstert.2004.08.042 16009168

[58] Ramos L Wetzels AM. Low rates of DNA fragmentation in selected motile human spermatozoa assessed by the TUNEL assay. Hum Reprod (2001) 16(8):1703–7. 10.1093/humrep/16.8.1703 11473968

[59] Piomboni P Bruni E Capitani S Gambera L Moretti E La Marca A Ultrastructural and DNA fragmentation analyses in swim-up selected human sperm. Arch Androl (2006) 52(1):51–9. 10.1080/01485010500203741 16338870

[60] Hoshi K Katayose H Yanagida K Kimura Y Sato A. The relationship between acridine orange fluorescence of sperm nuclei and the fertilizing ability of human sperm. Fertil Steril (1996) 66(4):634–9. 8816630 10.1016/s0015-0282(16)58581-1

[61] Liu DY Baker HW. Human sperm bound to the zona pellucida have normal nuclear chromatin as assessed by acridine orange fluorescence. Hum Reprod (2007) 22(6):1597–602. 10.1093/humrep/dem044 17369294

[62] Van Dyk Q Lanzendorf S Kolm P Hodgen GD Mahony MC. Incidence of aneuploid spermatozoa from subfertile men: selected with motility versus hemizona-bound. Hum Reprod (2000) 15(7):1529–36. 10.1093/humrep/15.7.1529 10875861

[63] Estop A Catala V Santalo J. Chromosome constitution of highly motile mouse sperm. Mol Reprod Dev (1990) 27(2):168–72. 10.1002/mrd.1080270213 2248781

[64] Schuster TG Cho B Keller LM Takayama S Smith GD. Isolation of motile spermatozoa from semen samples using microfluidics. Reprod Biomed Online (2003) 7(1):75–81. 10.1016/S1472-6483(10)61732-4 12930579

[65] Nosrati R Vollmer M Eamer L San Gabriel MC Zeidan K Zini A Rapid selection of sperm with high DNA integrity. Lab Chip (2014) 14(6):1142–50. 10.1039/c3lc51254a 24464038

[66] Berkovitz A Eltes F Yaari S Katz N Barr I Fishman A The morphological normalcy of the sperm nucleus and pregnancy rate of intracytoplasmic injection with morphologically selected sperm. Hum Reprod (2005) 20(1):185–90. 10.1093/humrep/deh545 15471930

[67] Bartoov B Eltes F Pansky M Langzam J Reichart M Soffer Y. Improved diagnosis of male fertility potential via a combination of quantitative ultramorphology and routine semen analyses. Hum Reprod (1994) 9(11):2069–75. 7868676 10.1093/oxfordjournals.humrep.a138395

[68] Greco E Scarselli F Fabozzi G Colasante A Zavaglia D Alviggi E Sperm vacuoles negatively affect outcomes in intracytoplasmic morphologically selected sperm injection in terms of pregnancy, implantation, and live-birth rates. Fertil Steril (2013) 100(2):379–85. 10.1016/j.fertnstert.2013.04.033 23706334

[69] Ebner T Shebl O Oppelt P Mayer RB. Some reflections on intracytoplasmic morphologically selected sperm injection. Int J Fertil Steril (2014) 8(2):105–12. 25083173 PMC4107682

[70] Ebner T Shebl O Moser M Mayer RB Arzt W Tews G. Easy sperm processing technique allowing exclusive accumulation and later usage of DNA-strandbreak-free spermatozoa. Reprod Biomed Online (2011) 22(1):37–43. 10.1016/j.rbmo.2010.09.004 21115273

[71] Enciso M Pieczenik G Cohen J Wells D. Development of a novel synthetic oligopeptide for the detection of DNA damage in human spermatozoa. Hum Reprod (2012) 27(8):2254–66. 10.1093/humrep/des201 22693169

[72] Sanchez V Redmann K Wistuba J Wubbeling F Burger M Oldenhof H Oxidative DNA damage in human sperm can be detected by Raman microspectroscopy. Fertil Steril (2012) 98(5):1124–9. 10.1016/j.fertnstert.2012.07.1059 22835447

[73] Parmegiani L Cognigni GE Bernardi S Troilo E Ciampaglia W Filicori M. Physiologic ICSI: hyaluronic acid (HA) favors selection of spermatozoa without DNA fragmentation and with normal nucleus, resulting in improvement of embryo quality. Fertil Steril (2010) 93(2):598–604. 10.1016/j.fertnstert.2009.03.033 19393999

[74] Huszar G Jakab A Sakkas D Ozenci CC Cayli S Delpiano E Fertility testing and ICSI sperm selection by hyaluronic acid binding: clinical and genetic aspects. Reprod Biomed Online (2007) 14(5):650–63. 10.1016/S1472-6483(10)61060-7 17509211

[75] Hamatani T Falco G Carter MG Akutsu H Stagg CA Sharov AA Age-associated alteration of gene expression patterns in mouse oocytes. Hum Mol Genet (2004) 13(19):2263–78. 10.1093/hmg/ddh241 15317747

[76] Junca A Gonzalez Marti B Tosti E Cohen M De la Fontaine D Benkhalifa M Sperm nucleus decondensation, hyaluronic acid (HA) binding and oocyte activation capacity: different markers of sperm immaturity? Case reports. J Assist Reprod Genet (2012) 29(4):353–5 10.1007/s10815-012-9710-5 22252415 PMC3309993

[77] Sato A Otsu E Negishi H Utsunomiya T Arima T. Aberrant DNA methylation of imprinted loci in superovulated oocytes. Hum Reprod (2007) 22(1):26–35. 10.1093/humrep/del316 16923747

[78] Titus S Li F Stobezki R Akula K Unsal E Jeong K Impairment of BRCA1-related DNA double-strand break repair leads to ovarian aging in mice and humans. Sci Transl Med (2013) 5(172):172ra21. 10.1126/scitranslmed.3004925 23408054 PMC5130338

[79] Genesca A Caballin MR Miro R Benet J Germa JR Egozcue J. Repair of human sperm chromosome aberrations in the hamster egg. Hum Genet (1992) 89(2):181–6. 10.1007/BF00217120 1587529

